# Efficacy of a Multi-lamellar Emulsion Containing a Synthetic Sphingosine Kinase 1 Activator and Pseudoceramide in Patients with Atopic Dermatitis: A Randomized Controlled Trial

**DOI:** 10.1007/s13555-024-01254-5

**Published:** 2024-08-30

**Authors:** So Yeon Lee, Jin Seo Park, Daehwan Kim, Wonseok Jeong, Chenghwan Hwang, Hye One Kim, Chun Wook Park, Bo Young Chung

**Affiliations:** 1 Department of Dermatology, Hallym University Kangnam Sacred Heart Hospital, Hallym University College of Medicine, Seoul, 07441 Republic of Korea; 2 CRID Center, NeoPharm Co., Ltd., Daejeon, Republic of Korea

**Keywords:** Atopic dermatitis, Moisturizer, Multi-lamellar emulsion, Pseudoceramide, Sphingosine kinase 1 activator, Stratum corneum hydration

## Abstract

**Introduction:**

Patients with atopic dermatitis (AD) have impaired barrier function, which decreases skin hydration, weakens their defense against microorganisms, and culminates in increased inflammatory responses. Here, we conducted a clinical trial to evaluate the efficacy of a multi-lamellar emulsion (MLE) containing the pseudoceramide PC-9S and a synthetic sphingosine kinase 1 (SPHK1) activator, Defensamide™, in improving mild-to-moderate atopic dermatitis.

**Methods:**

Forty patients aged ≥ 2 years were randomized into a combined-therapy group treated with the MLE containing PC-9S and Defensamide™ plus a topical corticosteroid and a topical-corticosteroid-only group. Assessments based on therapeutic methods included the Eczema Area and Severity Index (EASI), the Investigator Global Assessment (IGA), transepidermal water loss (TEWL), stratum corneum hydration (SCH), skin dryness, a visual analogue scale (VAS) of itchiness, a VAS of sleep disturbance, patient satisfaction, and the Dermatology Life Quality Index (DLQI).

**Results:**

Thirty-eight patients completed this study. In the combined-therapy group, significant improvements in clinical and instrumental measures such as EASI scores, skin hydration, and skin dryness were noted at 4 weeks compared to baseline, but such improvements were not noted in the topical corticosteroid-only group. Subjective assessments of itching and sleep disturbance and DLQI scores also showed significant improvements in the combined-therapy group.

**Conclusion:**

Combined therapy with the MLE containing Defensamide™ and PC-9S and with topical corticosteroid demonstrated superior clinical outcomes compared with topical corticosteroid monotherapy. Our findings underscore the potential of MLE-containing formulations as effective adjunctive therapies for AD, offering both objective and subjective symptomatic relief and enhancing patients' quality of life.

**Supplementary Information:**

The online version contains supplementary material available at 10.1007/s13555-024-01254-5.

## Key Summary Points

**Table:** 

This study evaluated the efficacy of a multi-lamellar emulsion (MLE) containing the pseudoceramide PC-9S and a synthetic sphingosine kinase 1 (SPHK1) activator, Defensamide™, in improving mild-to-moderate atopic dermatitis (AD).
Forty patients aged ≥ 2 years with AD were randomized into two groups: one treated with a combined therapy comprising the MLE containing PC-9S and Defensamide™ plus topical corticosteroid and the other treated with topical corticosteroid alone.
After 4 weeks, the combined-therapy group showed significant improvements in Eczema Area and Severity Index (EASI) score, skin hydration, subjective assessments of itching and sleep disturbance, and Dermatology Life Quality Index (DLQI) score.
Combined therapy with the MLE containing PC-9S and Defensamide™ plus topical corticosteroid demonstrated superior clinical outcomes and improved quality of life compared to corticosteroid monotherapy.

## Introduction

Atopic dermatitis (AD) is a prevalent inflammatory skin condition worldwide. It is characterized by intense pruritus and erythema and the formation of dry, scaly patches, often accompanied by exudation or crusting, which is indicative of chronic and recurrent skin inflammation [1]. It typically begins in early childhood and has a particularly high incidence in infants and young children [2]. According to epidemiological research, AD affects 15–20% of children and 1–3% of adults worldwide, highlighting its prevalence in various age groups [3, 4]. AD has profound socio-economic impacts and imposes a significant psychological burden on patients and their families. Economically, AD results in substantial healthcare costs due to the need for frequent medical visits, prescription medications, and hospitalizations as well as indirect costs from lost productivity and absenteeism at work or school [5]. The constant itching and visible skin lesions can cause embarrassment and self-consciousness, contributing to psychological distress, including anxiety and depression [6]. In particular, children with AD often experience bullying and social stigma, which can further exacerbate their emotional difficulties [7]. Moreover, AD affects patients' quality of life considerably, leading to social isolation and impairment of daily activities. These psychosocial challenges underscore the necessity of holistic treatment approaches that address both the physical and emotional needs of patients with AD, making the exploration of effective therapies even more pertinent.

The etiology of AD arises from complex interactions between genetic factors and environmental influences involving abnormalities in skin barrier function and immune system responses [8]. In individuals with AD, reduced levels of natural moisturizing factors (NMFs) and ceramides lead to skin dryness and increased transepidermal water loss (TEWL) [9]. These impairments in skin barrier function result in decreased skin hydration, weakened defense against microorganisms, and increased sensitization to allergens, culminating in heightened inflammatory responses [10]. Treatment for AD targets the restoration or maintenance of the skin barrier and diminishes inflammation and pruritus. Topical corticosteroids are used to treat acute flares as a first-line treatment for mild-to-moderate AD, whereas the daily application of emollients is advocated to manage and prevent AD lesions [11, 12].

Research has emphasized that the application of moisturizers containing lipid components can restore the skin barrier function in patients with AD [13, 14]. In particular, a multi-lamellar emulsion (MLE) containing a pseudoceramide known as PC-9S (the trade name for myristoyl/palmitoyl oxostearamide/arachamide MEA; Neopharm Co., Ltd., Daejeon, Korea), which closely mimics the multi-lamellar pattern of stratum-corneum intercellular lipids, was developed and subjected to clinical trials [15, 16]. A novel synthetic sphingosine kinase 1 (SPHK1) activator for keratinocytes, Defensamide™ (the trade name for (*S*)-methyl 2-(hexanamide)-3-(4-hydroxyphenyl) propanoate, MHP; Neopharm Co., Ltd.), has been shown to increase sphingosine-1-phosphate (S1P) levels, stimulate cyclic AMP production, and demonstrate the potential to reduce *Staphylococcus aureus* invasion in cultured human keratinocytes and human epidermal organotypic models, highlighting its role in promoting innate immunity [17]. However, the effectiveness of the SPHK1 activator Defensamide™ in patients with AD is yet to be determined.

The present study aimed to ascertain the efficacy of an MLE containing the pseudoceramide PC-9S as well as the SPHK1 activator Defensamide™ on AD, extending its scope to patients with AD aged ≥ 2 years, encompassing pediatric patients and beyond. Specifically, in real-world clinical practice, we evaluated the therapeutic efficacy of this MLE formulation in conjunction with topical corticosteroid versus topical corticosteroid alone to improve mild-to-moderate AD.

## Methods

### Study Design

This study was a prospective, observer-blinded, single-center, randomized controlled trial to evaluate the efficacy of a combined treatment comprising the MLE containing PC-9S and Defensamide™ and topical corticosteroid versus that of topical corticosteroid monotherapy for improving AD. This study was conducted in accordance with the principles outlined in the Declaration of Helsinki. Written informed consent was obtained from all participants, their parents, or guardians, and the study was approved by the institutional review board of Kangnam Sacred Heart Hospital (IRB no. 2021-10-023).

From November 2021 to October 2022, this study targeted patients diagnosed with AD who were aged ≥ 2 years and visited the Dermatology Outpatient Department of Hallym University Kangnam Sacred Heart Hospital. The inclusion criteria were as follows: (1) patients diagnosed with AD by a board-certified dermatologist based on the criteria established by Hanifin and Rajka [18]; (2) patients with mild-to-moderate severity of AD, as confirmed by board-certified dermatologists with an Eczema Area and Severity Index (EASI) score ≤ 20. Patients with clinical skin infections, concomitant uncontrolled systemic diseases, pregnant women, lactating women, or those deemed unsuitable for the study by the investigator were excluded. Within 4 weeks before or during the clinical trial period, the concomitant use of systemic corticosteroids, systemic immunomodulators, systemic antihistamines, and phototherapy was prohibited. The participants were not allowed to apply topical immunomodulators, topical corticosteroids, or topical antibiotics within 1 week before or during the study. This clinical trial randomized a total of 40 patients with mild-to-moderate AD who were aged ≥ 2 years into a combined-therapy group and a monotherapy group. The combined-therapy group received the study product (Zeroid® intensive rich cream, Neopharm Co., Ltd.) and topical corticosteroid (prednisolone valeroacetate 0.3%, Lidomex lotion, SamA Pharm. Co., Ltd., Wonju, Korea) for the first 2 weeks. Subsequently, for the next 2 weeks, the combined-therapy group continued with the study product alone. In contrast, the monotherapy group received topical corticosteroid for only the first 2 weeks. Additionally, within 5 days before and during the trial period, the use of any other moisturizer apart from the study product was prohibited. The major active ingredients of the products used in this study are listed in Supplementary Table 1.

In the combined-therapy group, the subjects applied the study product twice daily (morning and evening) to the entire body, starting from visit 1. For the affected skin areas, topical corticosteroid was applied to the skin lesions twice daily (morning and evening) for the first 2 weeks, followed by the application of the study product alone twice daily (morning and evening) for the subsequent 2 weeks. In the monotherapy group, topical corticosteroids were applied twice daily (morning and evening) to the affected skin areas for the first 2 weeks.

Participants visited the study site at 2 weeks (visit 2; day 14 ± 3) and 4 weeks (visit 3; day 28 ± 3) for clinical evaluation as well as subjective symptom assessment.

### Outcome Measurement

#### Eczema Area and Severity Index (EASI) and Investigator Global Assessment (IGA)

At visits 1, 2, and 3, the investigator assessed the patients’ EASI (range 0–72) and IGA scores (range 0–4). The EASI was calculated as a proportional factor ranging from 0 to 6 across four body regions: head and neck, lower limbs, upper limbs, and trunk. The EASI evaluates erythema (E), infiltration and/or population (I), excoriation (Ex), and lichenification (L) on a scale of 0–3. To calculate the EASI, the sum of the clinical sign scores (E + I + Ex + L) for each body region was multiplied by the body surface area and further multiplied by a proportional factor. The IGA was measured on a scale of 0–4, with 0 corresponding to clear, 1 to almost clear, 2 to mild, 3 to moderate, and 4 to severe [19].

#### Transepidermal Water Loss (TEWL) and Stratum Corneum Hydration (SCH)

The TEWL (g/m^2^/h) and SCH levels (arbitrary unit) were assessed at visits 1, 2, and 3 in all patients. Measurements were conducted using a VapoMeter (Delfin Technologies Ltd., Kuopio, Finland) and a moisturemeter (Delfin Technologies Ltd.). After cleansing the affected area and drying it thoroughly, measurements were taken by placing the probe in contact with the skin in an enclosed area at a temperature of 20–25 °C.

#### Skin Dryness

At visits 1, 2, and 3, the investigator assessed the scaling and roughness of the skin, while the participants recorded their feelings of dry skin and skin tightness. Each item was evaluated on a scale of 0–3 (0 = absent, 1 = mild, 2 = moderate, and 3 = severe).

#### Subjective Assessment of Itchiness, Sleep Disturbance, Satisfaction, and Dermatology Life Quality Index (DLQI)

At visits 1, 2, and 3, the patients directly recorded their subjective assessments of itching and sleep disturbance on a VAS ranging from 0 to 10. A score of 0 indicated no itchiness or sleep disturbance, whereas a score of 10 indicated the most severe symptoms. Additionally, at visit 3, both the combined-therapy and monotherapy groups were surveyed for their satisfaction with the treatment (very satisfied, satisfied, moderately dissatisfied, and very dissatisfied) and completed the DLQI [20]. The DLQI comprises 10 questions and is categorized into different subscales: symptoms and feelings (questions 1 and 2), daily activities (questions 3 and 4), leisure (questions 5 and 6), work and school (question 7), personal relationships (questions 8 and 9), and treatment (question 10). The DLQI score was computed by summing the scores from these 10 questions, with a maximum score of 30 and a minimum score of 0. A higher DLQI score indicates a greater impairment of quality of life.

### Adverse Events

Adverse events that could occur during the clinical trial were assessed at visits 2 and 3. These included the presence of adverse reactions such as erythema, swelling, itching, pain, or tingling.

### Statistical Analysis

Continuous variables were expressed as mean ± standard deviation. Repeated-measures analysis of variance (ANOVA) was conducted to analyze the changes observed in both groups over time. Independent-sample* t-*tests were used to compare the two groups at each time point. The efficacy of treatment before and after therapy was verified using paired* t*-tests or Wilcoxon signed-rank tests. Statistical significance was set at *p* < 0.05. All statistical analyses were performed using IBM SPSS Statistics (version 24.0; IBM Co., Armonk, NY, USA).

## Results

### Demographics

Forty subjects were enrolled in the study, with 20 assigned to the combined-therapy group and 20 to the monotherapy group through random allocation. In the monotherapy group, two subjects were lost to follow-up during the study period due to unavailability to contact and were consequently excluded, leaving 38 subjects who completed the study. At visit 1, there were no statistically significant differences in age, sex, EASI, IGA, SCH, TEWL, skin dryness, or DLQI scores between the two groups (Table 1).

**Table 1:** Baseline characteristics of the participants

	Combined-therapy group (*n* = 20)	Monotherapy group (*n* = 18)	Total (*n* = 38)	*p* value
Age	23.85 ± 22.02	28.33 ± 20.24	25.97 ± 21.03	0.519
Gender (male/female)	7/13	9/9	16/22	0.350
EASI score for head	0.29 ± 0.51	0.39 ± 0.58	0.33 ± 0.54	0.559
EASI score for upper extremity	1.45 ± 1.11	1.02 ± 1.16	1.25 ± 1.14	0.525
EASI score for trunk	0.95 ± 1.33	1.12 ± 1.87	1.03 ± 1.59	0.744
EASI score for lower extremity	1.90 ± 1.67	1.78 ± 2.87	1.84 ± 2.28	0.872
Total EASI score	4.58 ± 3.02	4.31 ± 5.48	4.45 ± 4.30	0.847
IGA	1.70 ± 0.60	1.60 ± 0.60	1.70 ± 0.60	0.645
TEWL	12.04 ± 8.76	12.51 ± 8.96	12.26 ± 8.80	0.819
SCH	30.85 ± 18.32	39.66 ± 23.07	35.02 ± 21.40	0.068
Skin dryness	7.90 ± 1.50	7.10 ± 1.40	7.50 ± 1.50	0.083
VAS for itching	5.20 ± 2.10	3.80 ± 1.50	4.60 ± 2.00	0.033*
VAS for sleep disturbance	2.70 ± 2.80	0.70 ± 0.70	1.70 ± 2.30	0.005*
DLQI	6.90 ± 4.80	5.30 ± 2.90	6.10 ± 4.10	0.231

*EASI* Eczema Area and Severity Index, *IGA* Investigator Global Assessment, *TEWL* transepidermal water loss, *SCH* stratum corneum hydration, *VAS* visual analogue scale, *DLQI* Dermatology Life Quality Index

*p < 0.05

Bold values indicate the statistically significant

However, significant differences were observed between the combined-therapy and monotherapy groups in the subjective assessments made by the subjects regarding itching and sleep disturbance (i.e., their VAS scores). For the itching VAS, the combined-therapy group reported a mean score of 5.20 ± 2.10, while the monotherapy group had a lower mean score of 3.80 ± 1.50 (*p* = 0.033). In the case of the sleep disturbance VAS, the combined-therapy group reported a mean score of 2.70 ± 2.80, whereas the monotherapy group had a lower mean score of 0.70 ± 0.70 (*p* = 0.005).

### EASI (Eczema Area and Severity Index) Score

**Fig. 1:**
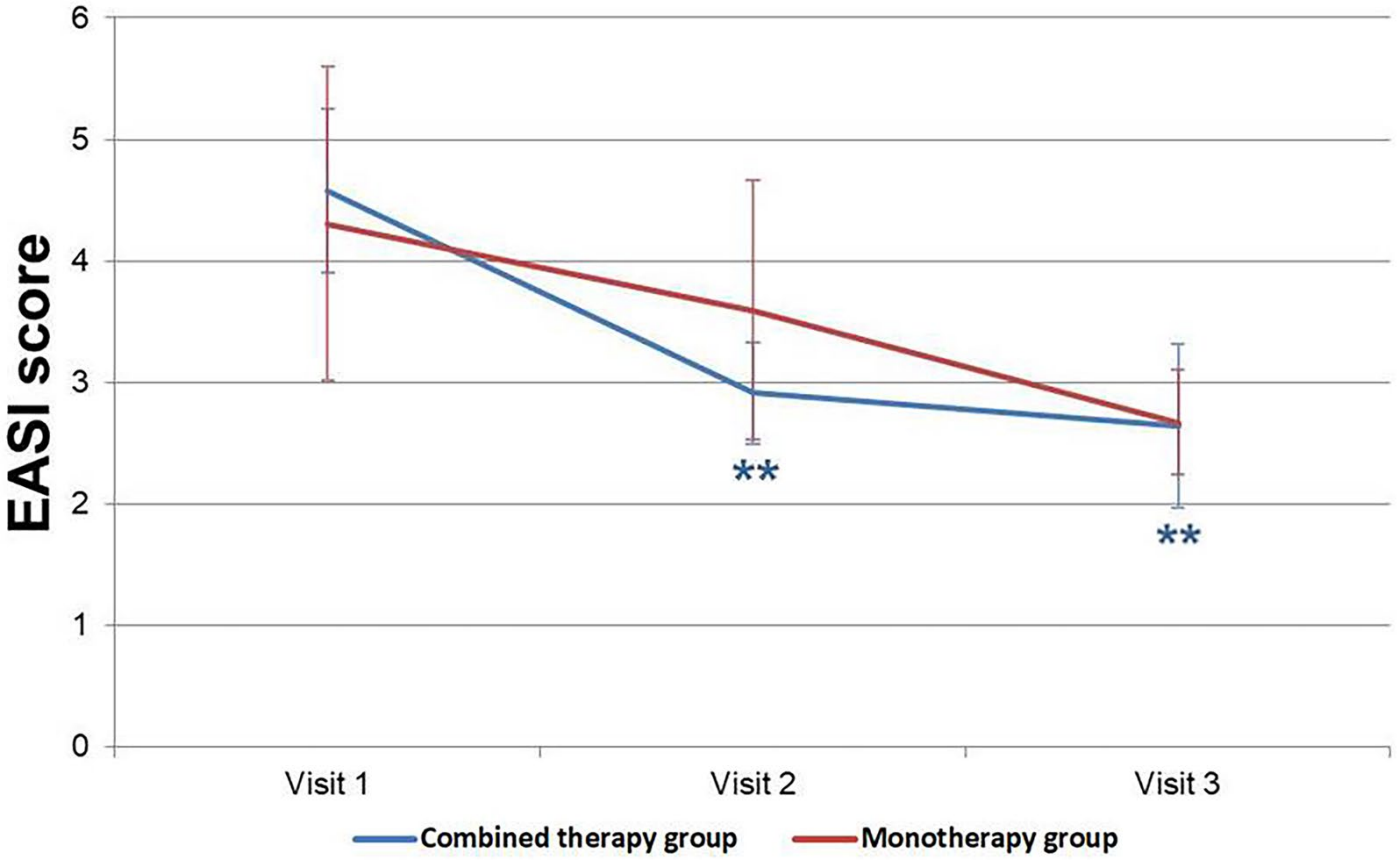
Changes in mean EASI (Eczema Area and Severity Index) score. Statistically significant decreases in score were observed in the combined-therapy group when visit 1 was compared to both visit 2 and visit 3 (visit 1–visit 2: *p* < 0.001; visit 1–visit 3: *p* = 0.002)

The EASI scores significantly decreased in the combined-therapy group at both visit 2 and visit 3 compared to visit 1 (visit 1–visit 2: *p* < 0.001; visit 1–visit 3: *p* = 0.002), with the scores improving from 4.58 ± 3.03, 2.92 ± 1.83, and 2.65 ± 2.95 at visits 1, 2, and 3. However, in the monotherapy group, there were no significant differences observed between the EASI scores at different visits, with scores changing from 4.31 ± 5.32 to 3.59 ± 4.39 to 2.67 ± 1.76 at visits 1, 2, and 3, respectively (Fig. 1).

We further subdivided the combined-therapy group and monotherapy group into individuals under 18 years of age (combined-therapy group: 11 individuals; monotherapy group: 9 individuals) and those 19 years or older (combined-therapy group: 9 individuals; monotherapy group: 9 individuals) for analysis. We found that a significant improvement was observed only in the EASI score of the combined-therapy group's child/adolescent subgroup between visits 1 and 3 (*p* = 0.001).

### IGA (Investigator Global Assessment)

**Fig. 2:**
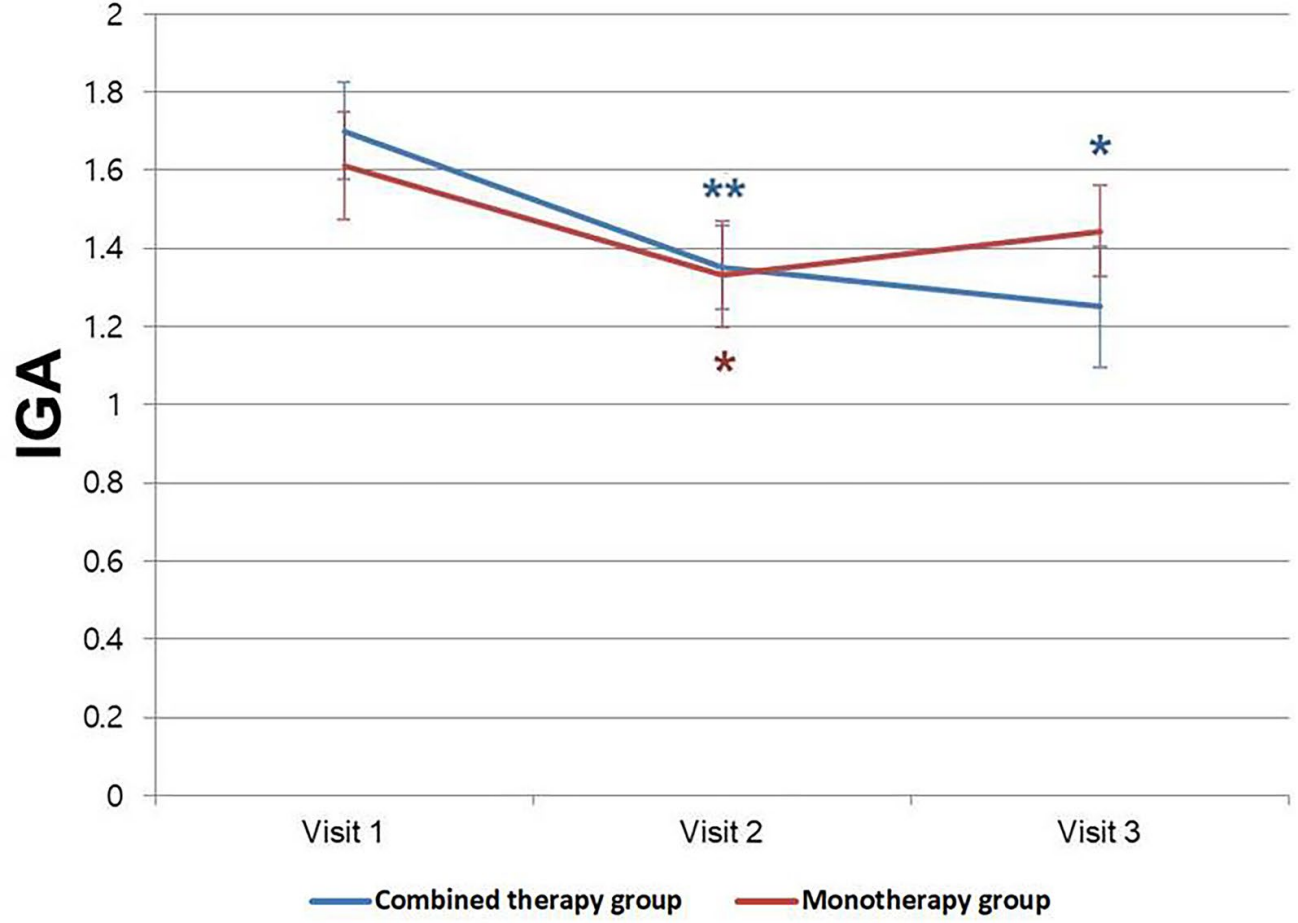
Changes in IGA (Investigator Global Assessment) score. Statistically significant decreases in score were observed in the combined-therapy group when visit 1 was compared to both visit 2 and visit 3 (visit 1–visit 2: *p* = 0.005; visit 1–visit 3: *p* = 0.016). In the monotherapy group, a statistically significant decrease was observed when visit 1 was compared to visit 2 (visit 1–visit 2: *p* = 0.021). Data were expressed as mean ± standard deviation

The combined-therapy group exhibited significant reductions in IGA scores at both visit 2 and visit 3 compared to visit 1 (visit 1–visit 2: *p* = 0.005, visit 1–visit 3: *p* = 0.016), with the scores improving from 1.70 ± 0.56 to 1.35 ± 0.48 to 1.25 ± 0.70 at visits 1, 2, and 3, respectively (Fig. 2). In the monotherapy group, there was a significant reduction in IGA scores only at visit 2 compared to visit 1 (visit 1–visit 2: *p* = 0.021), with scores improving from 1.61 ± 0.59 to 1.33 ± 0.58 to 1.44 ± 0.50 at visits 1, 2, and 3, respectively.

### TEWL (Transepidermal Water Loss) and SCH (Stratum Corneum Hydration)

**Fig. 3:**
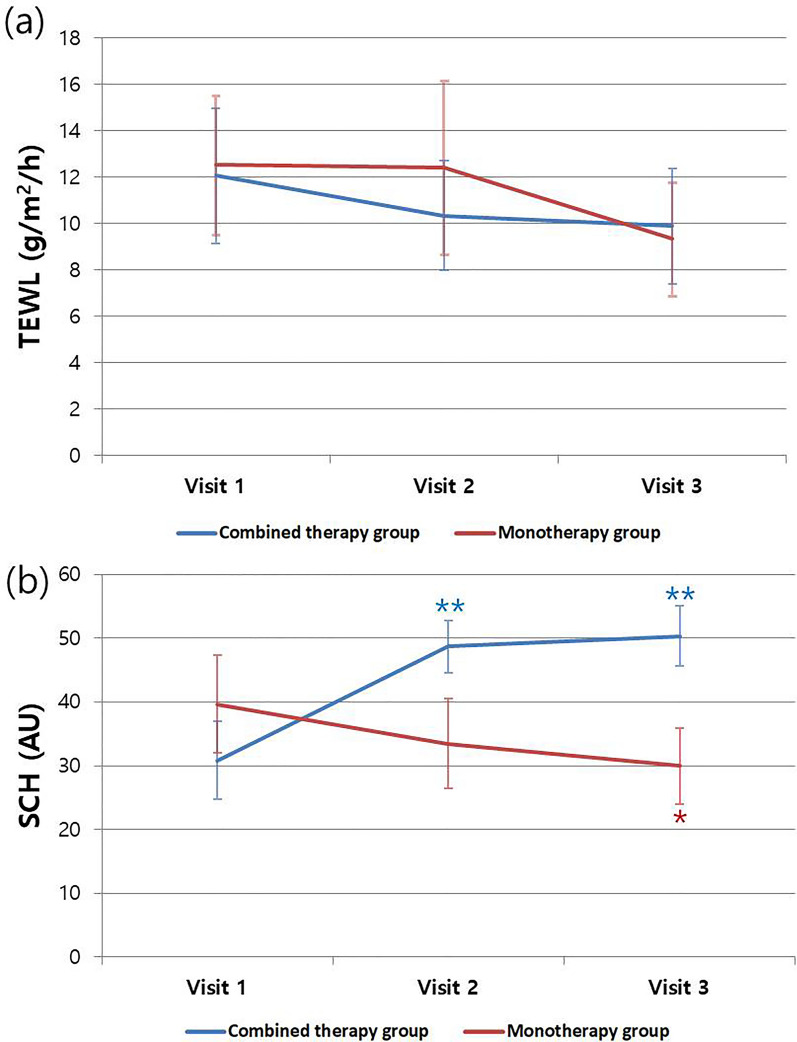
Changes in TEWL (transepidermal water loss) and stratum corneum hydration (SCH). **A** TEWL showed a decreasing trend, but no significant differences were observed in either group. **B** A statistically significant increase in SCH was noted for the combined-therapy group when visit 1 was compared to both visit 2 and visit 3 (visit 1–visit 2: *p* < 0.001; visit 1–visit 3: *p* < 0.001). On the contrary, a statistically significant decrease in SCH was observed for the monotherapy group when visit 1 was compared to visit 3 (visit 1–visit 3: *p* = 0.037)

To evaluate the changes in skin barrier function, we measured TEWL and SCH levels. TEWL levels at visits 1, 2, and 3 in the combined-therapy group were 12.04 ± 8.79, 10.33 ± 8.50, and 9.87 ± 6.02, respectively, while those in the monotherapy group were 12.51 ± 8.19, 12.40 ± 9.31, and 9.32 ± 5.43, respectively. TEWL showed a decreasing trend, but no significant differences were observed in either group (Fig. 3A).

The combined-therapy group exhibited a significant increase in SCH levels at both visit 2 and visit 3 compared to visit 1 (visit 1–visit 2: *p* < 0.001; visit 1–visit 3: *p* < 0.001). SCH levels in the combined-therapy group were 30.84 ± 19.97, 48.67 ± 15.96, and 50.31 ± 12.21 at visits 1, 2, and 3, respectively. In contrast, in the monotherapy group, SCH levels decreased gradually from visit 1 to visits 2 and 3. In particular, a significant decrease in SCH levels was noted at visit 3 compared with visit 1 (visit 1–visit 3: *p* = 0.037) (Fig. 3B). SCH levels in the monotherapy group were 39.66 ± 20.01, 33.50 ± 17.00, and 29.99 ± 11.06 at visits 1, 2, and 3, respectively. Furthermore, when comparing SCH levels between the two groups at visits 2 and 3, it was observed that the SCH level in the combined-therapy group was significantly higher than that in the monotherapy group at both visit 2 and visit 3 (visit 2: *p* = 0.008; visit 3: *p* < 0.001). When the results were stratified by age into under-18 and over-19 groups, there was a significant difference in SCH outcomes between visits 1 and 3 in the combined-therapy group, regardless of whether the participants were pediatric/adolescent or adult (children/adolescent group: *p* < 0.001; adult group: *p* = 0.012).

### Skin Dryness

**Fig. 4:**
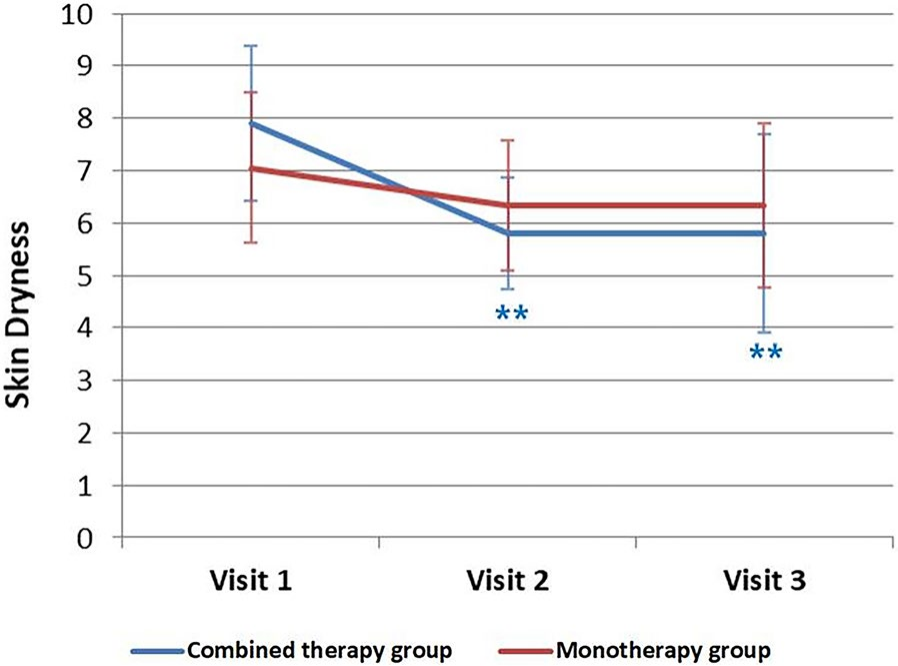
Changes in skin dryness. Statistically significant decreases were observed in the combined-therapy group when visit 1 was compared to both visit 2 and visit 3 (visit 1–visit 2: *p* < 0.001; visit 1–visit 3: *p* < 0.001)

The combined-therapy group demonstrated a significant decrease in skin dryness at both visit 2 and visit 3 compared to visit 1 (visit 1–visit 2: *p* < 0.001; visit 1–visit 3: *p* < 0.001) (Fig. 4). In the monotherapy group, no significant differences were observed in skin dryness between visits (visit 1–visit 2: *p* = 0.160; visit 1–visit 3: *p* = 0.3520). When we subdivided the combined-therapy group and the monotherapy group into individuals under 18 years of age and those 19 years of age or older, skin dryness showed significant differences between visits 1 and 3 in the combined-therapy group, irrespective of whether the participants were children/adolescent or adults (children/adolescent group: *p* < 0.011; adult group: *p* = 0.019).

### Itching and Sleep Disturbance VAS (Visual Analogue Scale)

**Fig. 5:**
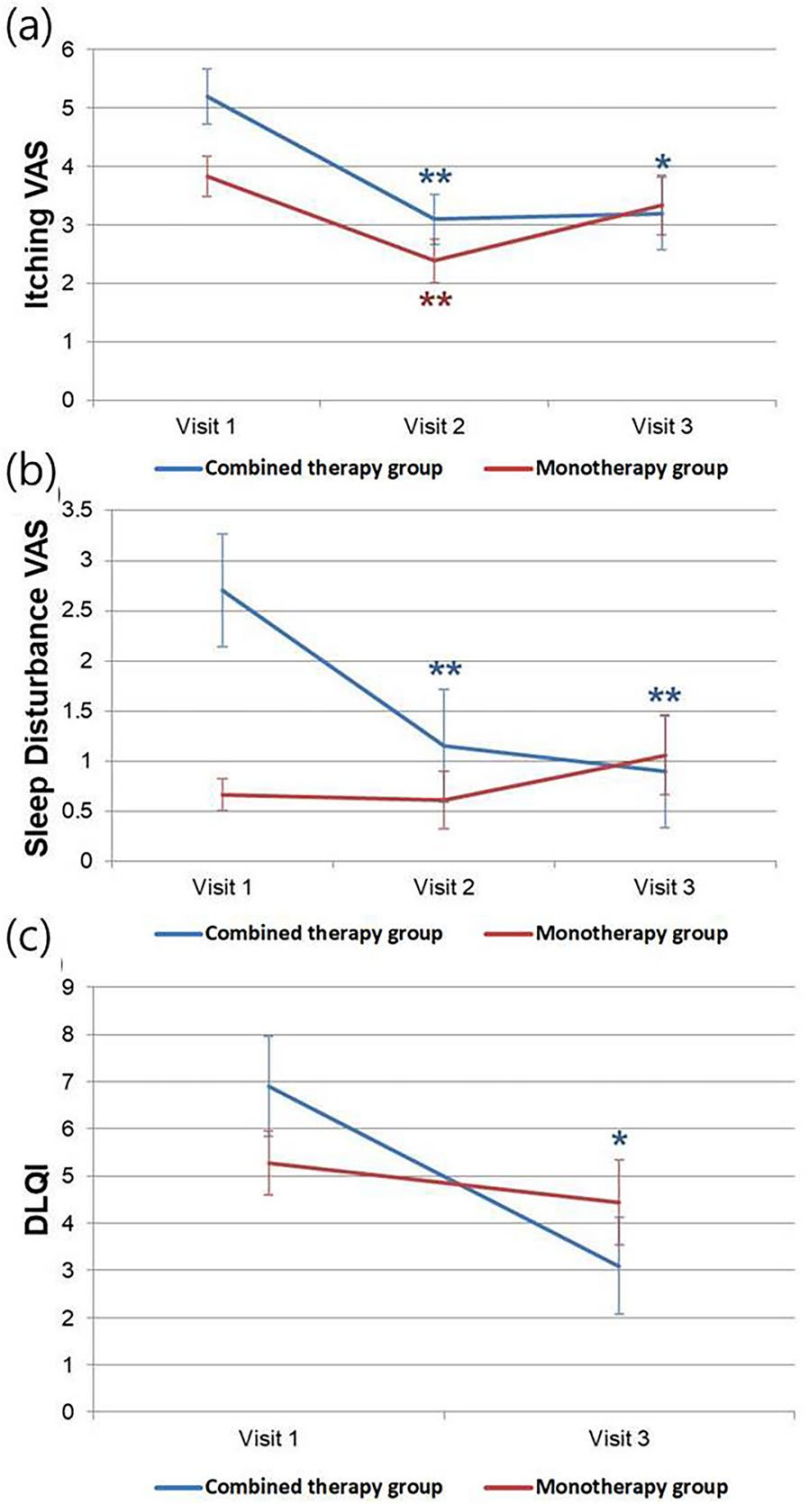
Changes in itching VAS, sleep disturbance VAS, and DLQI scores. **A** In the itching VAS, a statistically significant decrease in score was observed in the combined-therapy group when visit 1 was compared to both visit 2 and visit 3 (visit 1–visit 2: *p* = 0.001; visit 1–visit 3: *p* = 0.016) and in the monotherapy group when visit 1 was compared to visit 2 (visit 1–visit 2: *p* = 0.001). **B** For sleep disturbance VAS, a statistically significant decrease in score was only observed in the combined-therapy group when visit 1 was compared to both visit 2 and visit 3 (visit 1–visit 2: *p* = 0.001; visit 1–visit 3: *p* = 0.004). **C** DLQI showed a statistically significant decrease in the combined-therapy group when visit 1 was compared to visit 3 (*p* = 0.031). *VAS* Visual analogue scale, *DLQI* Dermatology life quality index

In addition to the physicians’ assessments, subjective assessments of itching (using a VAS), sleep disturbance (using a VAS), and DLQI were performed. The combined-therapy group exhibited a significant decrease in itching VAS scores at both visits 2 and 3 compared with visit 1 (visit 1–visit 2: *p* = 0.001; visit 1–visit 3: *p* = 0.016). Specifically, the itching VAS scores in the combined-therapy group were 5.20 ± 2.14, 3.10 ± 1.92, and 3.22 ± 2.78 at visits 1, 2, and 3, respectively. However, the monotherapy group showed a significant decrease only at visit 2 compared to visit 1 (visit 1–visit 2: *p* = 0.001) (Fig. 5A). In the monotherapy group, the itching VAS scores were 3.83 ± 1.46, 2.39 ± 1.57, and 3.33 ± 2.16 at visits 1, 2, and 3, respectively. In the case of the sleep disturbance VAS, the scores for the combined-therapy group were 2.78 ± 2.76, 1.15 ± 1.52, and 0.92 ± 2.26 at visits 1, 2, and 3, respectively. The combined-therapy group exhibited significant decreases in sleep disturbance VAS scores at visits 2 and 3 compared with visit 1 (visit 1–visit 2: *p* = 0.001; visit 1–visit 3: *p* = 0.004). However, in the monotherapy group, no significant differences in sleep disturbance were observed between visits (Fig. 5B). The sleep disturbance VAS scores of the monotherapy group were 0.67 ± 0.67, 0.61 ± 1.20, and 1.06 ± 1.68 at visits 1, 2, and 3, respectively. When we subdivided the combined-therapy group and monotherapy group into individuals under 18 years of age and those 19 years of age or older, a significant improvement was observed only in the sleep disturbance VAS score of the combined-therapy group's adult subgroup between visits 1 and 3 (*p* = 0.018).

### Dermatology Life of Quality Index (DLQI)

The change in the DLQI score was analyzed between visits 1 and 3 within each group. The combined-therapy group demonstrated a significant reduction (*p* = 0.031), whereas the monotherapy group did not exhibit any significant difference (*p* = 0.418) (Fig. 5C). Specifically, in the combined-therapy group, the DLQI scores were 6.97 ± 4.77 at visit 1 and 3.15 ± 4.60 at visit 3. In contrast, in the monotherapy group, the DLQI scores were 5.28 ± 2.88 at visit 1 and 4.44 ± 3.86 at visit 3. When we subdivided the combined-therapy group and monotherapy group into individuals under 18 years of age and those 19 years of age or older, a significant improvement was observed only in the DLQI of the combined-therapy group's child/adolescent subgroup between visit 1 and visit 3 (*p* = 0.044).

### Patient Satisfaction

At the final visit (visit 3), satisfaction with the clinical trial was measured in both the combined-therapy and monotherapy groups. The combined-therapy group reported a significantly higher satisfaction level, with a mean score of 4.35 ± 0.48, while the monotherapy group scored 3.50 ± 0.90 (*p* < 0.001).

### Adverse Events

No adverse events were observed during visits 2 and 3, and no other adverse reactions were reported by the participants throughout the study.

## Discussion

This study investigated the efficacy of a combined therapy comprising topical corticosteroid and the study formulation versus monotherapy with topical corticosteroid. The study findings underscore the superiority of the combined therapy over monotherapy in addressing symptoms and enhancing the quality of life among patients with AD. Across objective measures and subjective assessments, the combined-therapy group exhibited significant improvements, including enhanced SCH levels, reduced itching and sleep disturbance, and improved DLQI scores. While the monotherapy group showed some improvement in IGA and itching at visit 2, these effects were not sustained at visit 3. However, the combined-therapy group demonstrated consistent enhancement throughout all the evaluation periods, suggesting a more robust and enduring therapeutic effect.

Topical corticosteroids are commonly used as the first-line treatment for mild-to-moderate AD [21]. However, several studies have indicated that the long-term use of topical corticosteroids can have detrimental effects on the skin barrier [22, 23]. In our study, the combined therapy comprising topical corticosteroid and an MLE containing PC-9S and Defensamide™ was observed to significantly improve the SCH level and to maintain the improved SCH level even after the discontinuation of corticosteroids, whereas the monotherapy with topical corticosteroid showed a decrease in SCH level. These results highlight the potential of combined therapy as a comprehensive approach for effectively managing AD symptoms and improving overall objective and subjective measures.

In this study, we investigated the efficacy of an MLE containing the pseudoceramide PC-9S and the SPHK1 activator Defensamide™ in treating AD across various age groups. Our study focused on an MLE containing the pseudoceramide PC-9S, designed to mimic the structure of natural ceramides, while the MLE closely resembles the brick-and-mortar architecture of the normal skin barrier, thereby emphasizing its potential benefits [15, 16, 24–26]. Previous research has indicated that an MLE containing pseudoceramide PC-9S, when compared to a placebo, can more effectively restore the stratum-corneum lamellar structure in vitro, thereby contributing to the recovery of a compromised skin barrier [16]. The MLE containing pseudoceramide PC-9S specifically prevented skin barrier dysfunction and the formation of 1-deoxysphingosine, which serves as a novel biomarker for skin barrier dysfunction, in pathological conditions with skin barrier disruption in a 3-dimensional skin equivalent and in a mouse model [25]. Additionally, when applied to adolescent patients with AD, moisturizers containing the MLE with pseudoceramide PC-9S exhibited significant improvements in the Scoring Atopic Dermatitis (SCORAD) index, surpassing the outcomes of urea-containing control moisturizers [15]. Notably, unlike previous studies, this study utilized a formulation containing an MLE containing not only pseudoceramide PC-9S but also the SPHK1 activator Defensamide™. A novel synthetic SPHK1 activator promoted the epidermal differentiation of murine skin and keratinocytes [27]. Furthermore, a synthetic SPHK1 activator enhanced epidermal innate immunity via the ceramide metabolite S1P, which activates cathelicidin production in keratinocytes. A topical synthetic SPHK1 activator also significantly suppressed the invasion of virulent *Staphylococcus aureus* into murine skin explants [17]. AD is a chronic inflammatory condition characterized by disruption of the skin barrier and susceptibility to skin infections [10]. Thus, the MLE containing PC-9S and Defensamide™ may have superior therapeutic effects compared to topical corticosteroid monotherapy, which may be attributed to its ability to restore impaired skin barrier function and enhance innate immunity and the antimicrobial defense.

In this study, when different age groups, namely pediatric/adolescent and adult subgroups, were analyzed, the outcomes differed between the overall patient population and the respective groups. Significant improvements in SCH and skin dryness were observed regardless of age, mirroring the findings for the entire combined-therapy group. However, significant improvements in the EASI score and DLQI that were previously observed in the combined-therapy group were evident only in the pediatric/adolescent group, which may be more responsive to our novel MLE formulation containing pseudoceramide PC-9S and Defensamide™ that target skin barrier restoration and inflammation reduction.

A heterogeneity between children and adults with AD has been suggested; this may be due to differences in genetics, immunology, and environmental triggers [28]. Children with AD show downregulation of lipid biosynthesis and higher Th2/Th17 immune responses, whereas adults exhibit increased Th1 responses and more normalized skin barriers with age [29]. Th2 inflammation predisposes AD skin to *Staphylococcus aureus* infection by inhibiting antimicrobial peptides in the skin [30]. It is known that staphylococcal species in the nares are replaced by other lipophilic bacteria post-adolescence, which may be associated with a reduction in the incidence or severity of AD [31]. Defensamide™, in particular, enhances innate immunity and provides defense against *Staphylococcus aureus*, a common pathogen that exacerbates skin infections in AD patients [32]. This is especially relevant in pediatric AD, where children are more susceptible to skin infections due to their still-developing immune systems [33]. In contrast, the adult subgroup showed significant improvements, primarily in sleep disturbance scores, potentially reflecting the impact of psychological stressors, which are more prevalent in adult life and known to exacerbate AD symptoms. Thus, these differences in immune profile, skin microbiota composition, and environmental factors between age groups in AD may contribute to the observed variations in therapeutic efficacy [34].

Extensive studies have investigated the role of emollients in the management of AD, revealing that many proprietary emollients have demonstrated benefits [35]. Previous research has shown that the combination of moisturizers with an active topical treatment achieves better outcomes compared to the active topical treatment alone, consistent with the result of this study. Hanifin et al. demonstrated that adding a moisturizing regimen to desonide 0.05% lotion significantly improved clinical signs and symptoms of AD, such as erythema, dryness, and pruritus, compared to the use of desonide 0.05% lotion alone [36]. Simpson et al. found that the use of Cetaphil Restoraderm™ moisturizer in combination with topical corticosteroid accelerated the reduction in the severity of AD compared to topical corticosteroid alone [37]. Our controlled, randomized study found that this MLE formulation combined with low-potency topical corticosteroid showed superior efficacy against traditional corticosteroid monotherapy. The approach used here not only assessed the impacts of these therapies on typical AD symptoms such as erythema and scaling but also evaluated skin hydration, barrier function, and utilized subjective assessments, such as those of pruritus and sleep disturbance. Our findings highlight that the MLE formulation can enhance treatment outcomes beyond those of a conventional topical corticosteroid, providing a promising adjunctive therapy option. This study suggests that advanced emollient formulations may further optimize management strategies for mild-to-moderate AD by offering improved skin barrier restoration and symptom control.

This study has the limitations of a relatively small sample size and a brief study duration. Additionally, due to the single-center design, potential biases may arise, making it challenging to generalize the findings. Thus, further investigation involving a larger patient cohort with a multi-center design and a longer study period will be needed. Furthermore, the topical corticosteroid used in this study was in a lotion form rather than the more commonly used hydrating ointment or cream formulations, which may account for the lower efficacy observed in the monotherapy group. Additionally, a limitation of our study is the inability to determine which component of the trial formulation—the pseudoceramide PC-9S or the SPHK1 activator Defensamide™—was primarily responsible for the significant therapeutic effects observed in the combined-therapy group. This should be addressed in future studies to delineate the specific contributions of each component. Lastly, it would have been meaningful to compare the effects of corticosteroid plus the novel MLE containing the pseudoceramide PC-9S and the synthetic SPHK1 activator, Defensamide™, against those of a traditional emollient used in combination with corticosteroid. However, by comparing the novel MLE formulation containing pseudoceramide PC-9S and Defensamide™ used in combination with corticosteroid to corticosteroid monotherapy without moisturizer application, our study sought not only to evaluate the specific benefits of the novel MLE formulation containing pseudoceramide PC-9S and Defensamide™ but also to highlight the importance of moisturizer application itself.

## Conclusion

Our study showed that the application of the MLE containing the pseudoceramide PC-9S and SPHK1 activator Defensamide™ with topical corticosteroid significantly improved the skin barrier function, clinical symptoms, and quality of life of patients with AD across a wide age range. Our findings reaffirm the enhanced therapeutic effects of combining topical corticosteroid with moisturizers and the prolonged maintenance of therapeutic benefits when transitioning to exclusive moisturizer application after the discontinuation of topical corticosteroid in mild-to-moderate AD.

## Supplementary Information

Below is the link to the electronic supplementary material.

Supplementary file1 (PDF 15 KB)

## Data Availability

The datasets generated during and/or analyzed during the current study are available from the corresponding author on reasonable request.
