# Supplementary material for: Efficacy of a Multi-lamellar Emulsion Containing a Synthetic Sphingosine Kinase 1 Activator and Pseudoceramide in Patients with Atopic Dermatitis: A Randomized Controlled Trial
Source: Dermatol Ther (Heidelb). 2024 Aug 30;14(9):2591–605. doi: 10.1007/s13555-024-01254-5 (PMC11393266; doi:10.1007/s13555-024-01254-5)
Supplement: Supplementary file 1 — Supplementary file1 (PDF 15 KB) [file 13555_2024_1254_MOESM1_ESM.pdf]

Efficacy of Multi-Lamellar Emulsion-Containing Synthetic Sphingosine Kinase 1 Activator and Pseudoceramide in Patients with Atopic Dermatitis: A Randomized Controlled Trial

So Yeon Lee[1], Jin Seo Park[1], Dae Hwan Kim[2], Won Seok Jeong[2], Cheng Hwan Hwang[2], Hye One Kim[1], Chun Wook Park[1], and Bo Young Chung[1]

Author details

[1] Department of Dermatology, Hallym University Kangnam Sacred Heart Hospital, Hallym University College of Medicine, Seoul, Republic of Korea

[2] CRID Center, NeoPharm Co., Ltd., Daejeon, Republic of Korea

Correspondence

Bo Young Chung

Department of Dermatology, Hallym University Kangnam Sacred Heart Hospital, Hallym University College of Medicine, Seoul, 07441, Republic of Korea

Tel : +82-829-5221

Fax : +82-832-3237

Email : victoryby@naver.com

ORCID : 0000-0002-2795-0140

Supplementary Table 1. List of major components of the moisturizer. Information was provided by NeoPharm Co., Ltd, Daejeon, Korea. *INCI* international nomenclature of cosmetic ingredients

**Ingredient (INCI)**
Myristoyl/palmitoyl oxostearamide/arachamide MEA
Phytosterols
Stearic acid
Methyl caprooyl tyrosinate
Glycerin
Theobroma cacao (Cocoa) seed butter
Olea europaea (Olive) fruit oil
Tocopheryl acetate
Squalane
Madecassoside
Allantoin
Panthenol
Zinc gluconate
